# The Associations Between Attachment Insecurity and Compulsive Sexual Behavior Disorder or Problematic Pornography Use: The Mediating Role of Emotion Regulation Difficulties

**DOI:** 10.1007/s10508-024-02904-7

**Published:** 2024-06-19

**Authors:** Magdalena Wizła, Karol Lewczuk

**Affiliations:** Institute of Psychology, Cardinal Stefan Wyszynski University, Wóycickiego 1/3, 01-938 Warsaw, Poland

**Keywords:** Compulsive sexual behavior disorder, Problematic pornography use, Emotion regulation, Attachment anxiety, Attachment avoidance, ICD-11

## Abstract

Compulsive sexual behavior disorder (CSBD) was previously considered an attachment disorder, while emotion dysregulation was thought to potentially be a key characteristic of it. However, this theoretical model was not tested in previous empirical research. In our cross-sectional study, we tested whether emotional regulation (ER) difficulties can be adopted as an explanatory mechanism for the relationships between attachment avoidance and anxiety, as well as CSBD and its most prevalent behavioral presentation—problematic pornography use (PPU). Participants (*n* = 1002; *M*_age_ = 50.49 years, *SD* = 13.32; men: 50.2%) completed an online survey regarding the investigated variables. In mediation analyses, attachment avoidance and anxiety were treated as simultaneous predictors, ER difficulties as a mediating variable, with CSBD/PPU severity as dependent variables. Emotion regulation difficulties and attachment anxiety had a direct positive effect on both CSBD and PPU. The direct effect of attachment avoidance on PPU was non-significant, and significant for CSBD depending on the measure used. Moreover, all the relationships between both insecure attachment dimensions and CSBD/PPU symptom severity were at least partially mediated by ER difficulties. Our results corroborate the theoretical claim that ER difficulties may be a useful framework for explaining the impact of attachment insecurity on CSBD/PPU. Theoretical and practical implications of the findings are discussed.

**Supplementary Information:**

The online version contains supplementary material available at 10.1007/s10508-024-02904-7.

## Introduction

Compulsive sexual behavior disorder (CSBD) is conceptualized as the inability to control persistent urges, thoughts, and behaviors in the sexual domain (Kraus et al., 2018; World Health Organization [WHO], 2022a). Compulsive sexual behavior disorder was previously considered an attachment disorder (Flores, 2004; Gilliland et al., 2015; Zapf et al., 2008), while emotion dysregulation was thought to be an important characteristic of it (Gola et al., 2022; Lew-Starowicz et al., 2020). This manuscript aims to explain the link between attachment insecurity and CSBD symptoms by adopting an explanatory mechanism of emotion regulation (ER) difficulties.

The five diagnostic criteria of CSBD as proposed in the ICD-11 (WHO, 2022b) regard (1) preoccupation with the sexual domain to the point it leads to (2) negative consequences and (3) impairment in other areas of life. Moreover, an individual experiencing CSBD (4) will have undertaken unsuccessful attempts to refrain from or limit sexual behaviors and (5) continues engagement in sexual activities despite lack of or diminished sexual satisfaction. The most prevalent behavioral presentation of CSBD is problematic pornography use (PPU) (up to 80%, e.g., Kraus et al., 2018) which is characterized by excessive and uncontrollable pornography use that is frequently employed as a coping strategy to deal with difficult emotions (e.g., de Alarcón et al., 2019; Fernandez & Griffiths, 2021; Wéry & Billieux, 2017). Compulsive sexual behavior disorder extends beyond PPU and refers to other out-of-control non-paraphilic sexual behaviors encompassing, for example, uncontrollable masturbation, excessive use of paid sexual services, and numerous casual sexual partners.

Compulsive sexual behavior disorder may be used as an umbrella term for possible subtypes of the disorder characterized by different behavioral presentations with various types of sexual behaviors (see Antons & Brand, 2021). The hypersexual disorder was CSBD’s proposed predecessor but was finally not included in the fifth edition of the *Diagnostic and Statistical Manual of Mental Disorders* (DSM-5; APA, 2013). Compulsive sexual behavior has also been conceptualized and researched as, among others, sexual addiction (Carnes, 1983; Griffiths, 2019), sexual compulsivity (Coleman, 1987; Kalichman & Rompa, 1995), or sexual impulsivity (Barth & Kinder, 1987). For the sake of consistency, we refer to all its previous conceptualizations as CSBD in accordance with the current classification in ICD-11 (WHO, 2022a, 2022b).

**Fig. 1:**
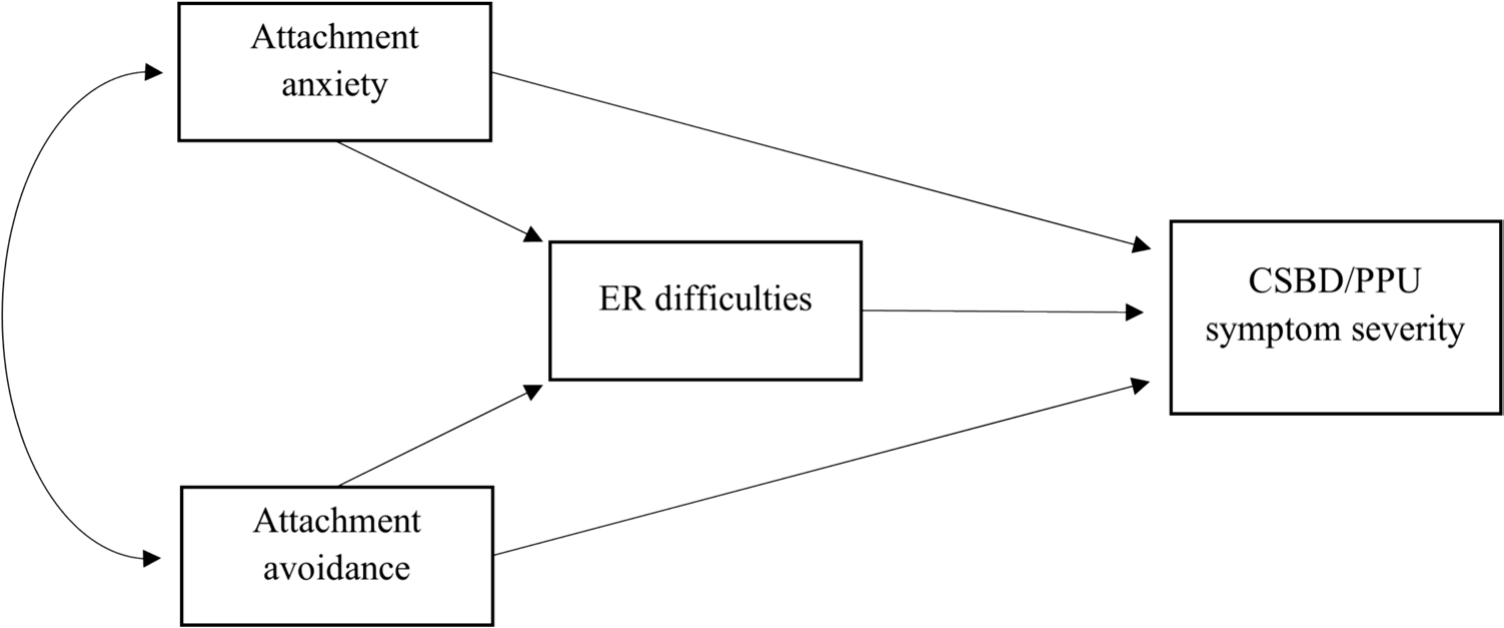
Conceptual diagram: mediation model of relationships between attachment anxiety, attachment avoidance, and compulsive sexual behavior disorder or problematic pornography use. ER—emotion regulation; CSBD—compulsive sexual behavior disorder; PPU—problematic pornography use

Although it was not ultimately included in the diagnostic criteria for CSBD, emotion dysregulation seems to potentially be an important factor in CSBD development and maintenance (see Gola et al., 2022; Lew-Starowicz et al., 2020) and has been postulated to be a core feature in previous conceptualizations of CSBD (e.g., Goodman, 1993; Kafka, 2010; Walton et al., 2017). The theory of attachment (Bowlby, 1977) posits that, during interactions with primary attachment figures, an individual develops a set of cognitive schemas about self and others, expectations, and strategies aimed at regulating proximity to others. Effective emotion regulation is one aspect of functioning shaped by the attachment figure’s responsiveness. The perception of emotion dysregulation as a product of insecure attachment styles leading to CSBD symptoms is suggested as a useful conceptual framework for understanding the disorder (Lew-Starowicz et al., 2020). In the current paper, we propose an empirically based model showing emotion regulation difficulties stemming from insecure attachment styles and how they contribute to CSBD and PPU symptom severity (see Fig. 1).

### Compulsive Sexual Behavior Disorder and Emotion Regulation

ER is defined as a process that an individual employs to influence the kind of emotions they feel and when, as well as how they experience and express them (Gross, 1998, 2002, 2014). The key role of emotion dysregulation in CSBD development and maintenance has been highlighted in its all major conceptualizations (Bancroft & Vukadinovic, 2004; Carnes, 1983; Goodman, 1993; Kafka, 2010; Walton et al., 2017). The diagnostic criteria of hypersexual disorder (CSBD’s predecessor, proposed for but ultimately not included in the DSM-5) included using sexual behaviors as a coping strategy, i.e., engaging in sexual activities in response to dysphoric mood states and stressful life events (Kafka, 2010). The current conceptualization of CSBD, as well as the instruments used for its assessment, is based on the ICD-11 criteria (WHO, 2022a) that do not include this criterion. Whether emotion dysregulation should be a diagnostic criterion for CSBD is, however, still under discussion (Gola et al., 2022).

Although the role of ER difficulties in CSBD is highlighted in the theoretical models, the empirical data on their associations is limited. It has been suggested that proneness to negative affect and difficulty in identifying and managing emotions can lead to employing hypersexual behavior as a coping mechanism in men seeking help for CSBD (Reid et al., 2008). For men with CSBD maladaptive coping strategies were adopted more frequently (Engel et al., 2019), while maladaptive shame coping predicted the severity of CSBD (Reid et al., 2011) which supports the hypothesis that CSBD can be used as an ER method. Also, among patients with substance use disorder (Hashemi et al., 2018) and highly sexually active men who have sex with men (MSM) (Pachankis et al., 2014), difficulties with ER were positively associated with hypersexual symptoms. The literature supports the existence of significant relationships between ER difficulties and CSBD, however, it is important to emphasize that in our study search, we did not come across data showing how ER difficulties relate to CSBD in female samples.

### Attachment and Emotion Regulation

According to Bowlby’s (1977) theory attachment is an instinct that can be described as “any form of behaviour that results in a person attaining or retaining proximity to some other differentiated and preferred individual” (p. 204). The most distinctive dimension characterizing attachment is not its strength but security vs. insecurity in the relationship (Ainsworth et al., 2015). Attachment anxiety and avoidance are two separate dimensions of attachment insecurity that determine different attachment behaviors (Brennan et al., 1998; Fraley et al., 2000). Attachment avoidance is associated with the fear of intimacy, interdependence, and emotional openness; while attachment anxiety is more strongly related with the fear of insufficient love or abandonment (Crowell et al., 2008). In line with this, avoidant attachment is related to deactivating ER strategies to alleviate distress resulting from frustration in interactions with a distant or rejecting attachment figure (Shaver & Mikulincer, 2007). On the other hand, anxious attachment is associated with hyperactivating ER strategies that develop as a way of catching the attention of the attachment figure to obtain soothing. Avoidantly attached individuals aim to dissociate from their emotions and are less focused on them which results in difficulties identifying emotions, while anxiously attached individuals are more aware of their emotions, but also have problems with clarifying them (Stevens, 2014). Moreover, anxious attachment was more often associated with engaging in impulsive behaviors and emotional interference with goals.

### Attachment Insecurity and Compulsive Sexual Behavior Disorder

Addiction may be considered a key symptom of attachment disorder (Flores, 2004). The same hypothesis was put forward for CSBD (e.g., Gilliland et al., 2015; Riemersma & Sytsma, 2013; Zapf et al., 2008) which could be perceived as a maladaptive strategy of affect regulation (Katehakis, 2009). Emotion regulation difficulties, in turn, stem from insecure attachment which is shaped by the inappropriate responsiveness of the caregiver (Katehakis, 2009).

A wide body of empirical evidence supports the existence of significant relationships between attachment insecurity and a higher possibility as well as a greater severity of CSBD (shared variance from 5 to 21%; see Efrati et al., 2022). A study on adolescents who presented CSBD symptoms showed that they significantly more often presented attachment anxiety (Efrati & Amichai-Hamburger, 2021; Efrati & Gola, 2018). As much as 95% of individuals diagnosed with sexual addiction showed characteristics of an anxious or avoidant attachment (Leedes, 1999a; Zapf et al., 2008). Men who sought treatment for CSBD reported secure attachment less frequently, while insecure styles were reported more often (Gilliland et al., 2015). Attachment anxiety and avoidance were also positively correlated with sexual compulsivity (Weinstein et al., 2015) and predicted hypersexual behavior for both heterosexual and homosexual men and women (Ciocca et al., 2021). Notably, the difference was predominantly explained by the factor reflecting the use of sexual behavior as a coping strategy for difficult emotions. Moreover, anxious attachment was a more powerful predictor of CSBD than the frequency of online and offline sexual activity (Efrati & Gola, 2018). Research remains inconsistent regarding which attachment insecurity dimension is a more powerful predictor of CSBD, with some pointing to avoidance (e.g., Crocker, 2015) and some to anxiety (e.g., Giordano et al., 2017; Kircaburun et al., 2023). Findings by Faisandier et al. (2012) point to a stronger association between attachment and out-of-control sexual behaviors in women than in men.

#### Anxious and Avoidant Attachment Associations with Compulsive Sexual Behavior Disorder

Both attachment dimensions exhibit distinct characteristics; however, they both positively contribute, as outlined in our literature review, to the severity of CSBD and PPU. The precise nature of the mechanisms underlying this phenomenon—where different characteristics predict the same disorder—remains understudied. To the best of our knowledge, existing research does not permit definite conclusions based on empirical data to be drawn in this field. The approach could be twofold: two divergent paths leading through different mechanisms to the same behaviors, or it may be possible that different dimensions predict various behavior patterns (Varfi et al., 2019). The associations may also be explained by the type of needs that the sexual behavior serves to satisfy; attachment anxiety was associated with fulfilling emotional needs, while attachment avoidance with sexual ones (Saint‐Eloi Cadely et al., 2020).

Individuals high in attachment anxiety are characterized by hyperregulating ER strategies leading to the intensification of negative emotions (Crowell et al., 2008). Such escalation of negative affect may lead to more frequent casual encounters in search of closeness or intimacy to ease them. However, casual sexual encounters may lack closeness which could be interpreted as rejection by an anxious individual, which would further intensify the difficult emotions in line with the hyperregulation assumption. Another explanation for engagement in casual sexual encounters may be an attempt to upregulate positive emotions. On the other hand, individuals high in attachment anxiety could engage in solitary sexual activity to cope with the difficult emotions that have been hyperregulated. Another reason for engaging in such activities could be to cope with feelings of abandonment and loneliness (see Mestre-Bach & Potenza, 2023).

A person high in attachment avoidance is characterized by emotion hyporegulation mostly employing deactivating ER strategies (Crowell et al., 2008). They fear intimacy and avoid bonding with others (Samenow, 2010; Weinstein et al., 2015; Zapf et al., 2008). It would mean that they might engage in casual sexual encounters to avoid forming intimate bonds or as a substitute for a close relationship (Leedes, 1999b, 2001). Pornography use or masturbation may serve the same purpose. For instance, teenagers who only engaged in sex online were higher in avoidance than those who engaged in both online and offline activities (Efrati & Amichai-Hamburger, 2021). Another reason for pornography use may be to escape difficult emotions (de Alarcón et al., 2019).

As explained above, the possible pathways that explain the associations between the two dimensions of insecure attachment and CSBD are manifold and complex. It seems that these relationships can be explained by studying the underlying mechanisms because, on the surface level both dimensions can be linked to the same behaviors (as also indicated by the literature review, showing no unequivocal differences in the predictive strength of anxiety and avoidance in relation to CSBD). The explanation of these associations needs to be disentangled in the future, based on further, more complex studies exploring the discussed mechanisms. However, this is beyond the scope of the current paper.

### Present Study

The literature review provides evidence for the associations of attachment styles, ER difficulties, and the development of CSBD and PPU. In addition, attachment theory is postulated to be a useful conceptual framework for explaining emotion dysregulation and the development of CSBD (see Lew-Starowicz et al., 2020), while CSB/PPU used as a means of coping with negative emotions and stress is considered to be a possible important characteristic of CSBD (Gola et al., 2022). Despite the data pointing to possible interconnections, to the best of our knowledge, no research has studied the formerly mentioned relationships between attachment insecurity, and CSBD or PPU symptom severity by employing ER difficulties as the mechanism explaining the links between insecure attachment and CSBD/PPU. In our study, we aimed to investigate how attachment insecurity (both anxiety and avoidance separately) predicts CSBD and PPU and how those relationships are mediated by ER difficulties. Next, as the current criteria for CSBD were recently introduced (WHO, 2022a), research on the assessment of and screening for the disorder symptoms is also in its initial phases, and comparative research juxtaposing available CSBD screening methods is lacking. Another issue is the possible overpathologization of normative sexual behavior (Billieux et al., 2015) as the existing measures yield presumably overestimated rates of people at high risk of CSBD/PPU (e.g., Lewczuk et al., 2023b). Due to this, a secondary aim of our investigation was to investigate whether the results obtained with two recently developed instruments assessing CSBD symptoms, that is, Compulsive Sexual Behavior Disorder-Diagnostic Inventory (CSBD-DI; Grubbs et al., 2023) and Compulsive Sexual Behavior Disorder Scale (CSBD-19; Bőthe et al., 2020a), are equivalent (or the results regarding the relationships between attachment and CSBD depend on the measure used). Both instruments were developed relatively recently and neither of them has a long-standing history of research supporting its reliability and validity. They are the only existing assessment tools based on the prevailing criteria for CSBD in accordance with ICD-11 (WHO, 2022b). However, there are very significant differences in the structure of scales, which reflect underlying differences in how these scales approach CSBD symptoms. The measures differ in the proportion of items reflecting each criterion which means a different weight is given to each criterion in CSBD-19 and CSBD-DI; as a result, specific criteria influence the overall score of the questionnaire differently. Another distinctive feature of the questionnaires is the scoring method: CSBD-19 (Bőthe et al., 2020a) employs a Likert scale that reflects the intensity of symptoms in the past 6 months, whereas CSBD-DI (Grubbs et al., 2023) only considers the fulfillment of the criterion during the same timeframe. Comparative studies of assessment instruments are crucial not only in a clinical context but also in research. This is important as it enables the simplification of procedures, taking into account respondents’ limited attention span, if both measures yield comparable results. Moreover, taking into account the relatively early stage of development of the two instruments, by comparing the outcomes yielded by these two scales, we can ensure that the obtained results do not depend on the specific scale used to assess CSBD, but are due to the underlying severity of CSBD symptoms. Thus, comparative research juxtaposing the two scales is of vital importance.

Not only is it important to contribute to existing knowledge by explaining the mechanisms behind previously studied relationships, but also by creating a mediation model that combines them in one framework. This approach provides a more comprehensive perspective that has previously only been theorized (Lew-Starowicz et al., 2020). These relationships pertain to difficulties with emotion regulation, attachment insecurity, and symptoms of PPU/CSBD. Additionally, in light of the replication crisis, especially in clinical psychology (Tackett & Miller, 2019; Tackett et al., 2019), and the field of behavioral addictions investigations (Eben et al., 2023), replicating the results is crucial. This is particularly important as, to our knowledge, our study is the first regarding attachment insecurity, and CSBD and PPU symptoms conducted in the Polish cultural context. Consequently, its significance results from the dependence of CSBD and PPU symptoms on cultural factors (Bőthe et al., 2023). Not only are attachment (Keller, 2013; Rothbaum et al., 2000; van Ijzendoorn & Sagi-Schwartz, 2008) and emotion regulation abilities (Keller & Otto, 2009; Matsumoto et al., 2008) sensitive to cultural differences in socialization, but also relationships between attachment, emotion regulation, and psychopathological symptoms can differ in various cultural contexts (Vatan & Pellitteri, 2016; Wang et al., 2022).

Based on the current knowledge, we hypothesize that both dimensions of insecure attachment, anxiety and avoidance, will have a direct positive effect on CSBD (effect of anxiety—Hypothesis 1 (H1); avoidance—H2) and PPU (anxiety—H3; avoidance—H4) symptom severity. Moreover, we presume that ER difficulties will have a direct positive effect on CSBD (H5) and PPU (H6) symptom severity. Another group of hypotheses regards the significant mediation of the relationship between attachment insecurity and CSBD and PPU symptom severity by difficulties with ER, at least a partial mediation of the relationship between attachment anxiety and CSBD (H7) as well as PPU (H8) by ER difficulties, and at least a partial mediation of the relationship between attachment avoidance and CSBD (H9) as well as PPU (H10) by ER difficulties.

As the likelihood of being affected by CSBD and PPU, their symptom severity and presentation can differ depending on age (e.g., Grubbs et al., 2019; Lewczuk et al., 2023b, 2023c), gender (e.g., Lewczuk et al., 2017; see also Kowalewska et al., 2020, 2022), sexual orientation (Edmundson et al., 2018), relationship status (e.g., Kumar et al., 2021; Lewczuk et al., 2023c), or the frequency of sexual behaviors (e.g., Chen et al., 2022; Lewczuk et al., 2023a, 2023b; see also Bőthe et al., 2020a, 2020b), we decided to include these variables in our analyses.

#### How Emotion Regulation Difficulties Can Mediate the Relationship Between Compulsive Sexual Behavior Disorder/Problematic Pornography Use Symptoms and Attachment Insecurity

Based on the literature review, attachment appears to be a preceding predisposition, shaped earlier in life, that can influence emotion regulation strategies in later life. Specifically, within its theoretical framework, attachment development is associated with learning strategies that regulate proximity to others (Bowlby, 1973, 1977). Interaction with significant others and their specific responses can shape the way a child regulates their emotions to facilitate bonding with the attachment figure. Attachment, being a construct with emotional components, naturally aligns closely with regulatory strategies and attachment theory is claimed to be one of the most significant theories explaining affect regulation (Mikulincer et al., 2003). The self-regulation model expands on the theory proposed by Bowlby (1973, 1977) by integrating it with dialectical philosophy and presents addiction as an impairment of self-regulation (Padykula & Conklin, 2010). Moreover, longitudinal studies indicate that early experiences of children with their caregivers influence their emotional abilities in adolescence (Fletcher et al., 2015). Infants' attachment predicts attachment-relevant emotion regulation strategies (Girme et al., 2021) and underlying neural processes during the regulation of positive emotions (Moutsiana et al., 2014) in adulthood. The seemingly developmentally preceding nature of attachment, serving as a learning environment for the development of specific ER difficulties (Shaver & Mikulincer, 2007; Stevens, 2014), justified the inclusion of ER difficulties as a mediator in our models.

Moreover, the mediating role of problems with ER in the relationship between attachment insecurity and psychopathological symptoms has been shown for most major psychiatric symptom classes: depressive symptoms (see Malik et al., 2015), PTSD severity (Benoit et al., 2010), binge eating behaviors (Shakory et al., 2015), or vegetative, agoraphobic, social phobia symptoms and global symptom severity index (Lewczuk et al., 2021a, 2021b). Both the relationship (1) between the independent variable (attachment avoidance or anxiety) and the mediator (ER difficulties), as well as (2) between the mediator (ER difficulties) and the dependent variable (CSBD or PPU symptom severity) have a very strong grounding in the literature—thus, a mediation model and not a moderation model was evaluated. However, the results need to be treated with caution because the reported data stems from cross-sectional and longitudinal studies. The moderating nature of ER difficulties may also be possible; for example, adaptive ER strategies were found to moderate the relationship between attachment and behavioral addictions in adolescents, while maladaptive strategies mediated it (Estevez et al., 2019). Therefore, as a part of additional analyses, we analyzed moderation models in which ER difficulties were placed in the role of a moderator of attachment—CSBD/PPU relationships. The results showed that none of the tested moderation effects were significant in any of the analyzed models (see Supplementary material).

## Method

### Participants and Procedure

The study was conducted via the Pollster research platform (https://pollster.pl/). Users registered on the platform participate in different studies collecting points that can later be exchanged for money or donated to a chosen organization (PanelBook 2023, 2024). All the measures included in our survey and general study aims have been preregistered and are available under the following link: https://osf.io/ywceq. Moreover, two questions checking the attention of participants were included in the survey and had to be answered correctly for the responses to be accepted. We received from the external data provider dataset comprising 1003 participants who completed all the measures relevant to our study—as a result, we have no knowledge about how many participants started (clicked the survey link) but did not complete the study. We excluded from the analyses one participant, as they did not specify their gender, resulting in a final sample of 1002 participants. Data was collected from June to July 2022. All the participants had previously taken part in one of two other studies from preceding years (2019 and 2020) conducted by our team.

The investigated sample includes 1002 Polish participants (*M*_age_ = 50.49 years, *SD* = 13.32), 503 of which were men (*M*_age_ = 51.71, *SD* = .56) and 499 women (*M*_age_ = 49.25, *SD* = 13.87). In terms of sexual orientation, the majority of participants (92.71%; *n* = 929) identified themselves as heterosexual, 3.19% (*n* = 32) as bisexual, 2.89% (*n* = 29) as homosexual, 0.20% (*n* = 2) as asexual, 0.10% (*n* = 1) as pansexual, and 0.09% (*n* = 9) chose the response option “other”. Regarding romantic relationships, the majority of participants (59.18%; *n* = 593) reported being in a formal relationship (married), 17.96% (*n* = 180) were in an informal relationship, and 22.85% (*n* = 229) were single. Participants were asked to complete a set of online measures regarding sociodemographic characteristics, attachment anxiety and avoidance, emotion regulation difficulties, CSBD, and PPU symptom severity.

### Measures

The Compulsive Sexual Behavior Disorder Scale (CSBD-19; Bőthe et al., 2020a, Polish adaptation: Bőthe et al., 2023) was used to assess the severity of CSBD symptoms. It is a 19-item (*α* = .94; *ω* = .94) scale with response options for each item ranging from 1 (Completely disagree) to 4 (Completely agree); sample item: I did not accomplish important tasks because of my sexual behavior. The general score was obtained from the sum of the items.

The Compulsive Sexual Behavior Disorder-Diagnostic Inventory (CSBD-DI; Original instrument and Polish adaptation: Grubbs et al., 2023) was another measure that we used for CSBD symptom severity assessment. It is a 9-item scale (*α* = .82; *ω* = .83) with three response options: two scored 0 (This has been true in my lifetime but not during the last 12 months and This has never been true of me), and one scored 1 (This has been true for at least 6 months during the last 12 months); sample item: I often engage in sexual behavior despite the risk of physical harm (e.g., sexually transmitted infection, unintended pregnancy, injury, or illness, etc.). The general score denoting the severity of CSBD symptoms was derived from the sum of responses from the first to seventh item (*α* = .76; *ω* = .77).

The Brief Pornography Screen (BPS; Kraus et al., 2020; Polish adaptation: Bőthe et al., 2023) was employed for the assessment of PPU symptom severity. The tool entails five items (*α* = .86; *ω* = .87) with the following response scale: 0 (*Never*), 1 (*Sometimes*), and 2 (*Frequently*); sample item: You continue to use pornography even though you feel guilty about it. The general score was calculated as the sum of the items.

The Experiences in Close Relationships-Revised (short version) (Fraley et al., 2000; Polish adaptation: Lubiewska et al., 2016) was used to measure insecure attachment dimensions: attachment anxiety and avoidance. The scale comprises 16 items with a response scale from 1 (*strongly disagree*) to 7 (*strongly agree*). The questionnaire has two 8-item subscales (1) attachment anxiety (*α* = .93; *ω* = .93), sample item: I often worry that my partner will not want to stay with me, (2) attachment avoidance (*α* = .89; *ω* = .89), sample item: I prefer not to be too close to romantic partners. The scores of the subscales were calculated as means of the items.

The Difficulties in Emotion Regulation Scale-Short Form (DERS-SF; Kaufman et al., 2016; Polish experimental version by Paweł Holas) is an instrument for the measurement of difficulties with emotion regulation we used. The scale is the short 18-item (*α* = .90; *ω* = .95) version of The Difficulties in Emotion Regulation Scale (DERS; Gratz & Roemer, 2004).^1^ It measures difficulties referring to 6 domains: nonacceptance of emotional responses, difficulty engaging in goal-directed behavior, impulse control difficulties, lack of emotional awareness, limited access to ER strategies, and lack of emotional clarity. The response scale ranged from 1 to 5 (1—*Almost Never* to 5—*Almost always*); sample item: When I’m upset I take time to figure out what I’m really feeling. In our analyses, we used only the general score expressed as the mean of all the items.

Participants were also asked to indicate the frequency of various sexual activities in the past 12 months: pornography use, masturbation, and sexual intercourse (Grubbs et al., 2019; Lewczuk et al., 2021a, 2021b; Wizła et al., 2022). The responses ranged from 0 to 7 (0—*never* to 8—*once a day or more often*).

We also adjusted for participants’ age, gender (dichotomous variable: 0—woman, 1—man), sexual orientation (coded dichotomously: 0—sexually diverse, 1—heterosexual), and relationship status (2 dummy variables with single being the reference group: in an informal relationship and a formal relationship [married]).

Reliability coefficients for all the used measures presented acceptable values of Cronbach's alpha and McDonald's omega (McDonald, 1970; McNeish, 2018; Nunnally, 1978). Apart from adequate coefficient values for CSBD-DI (Grubbs et al., 2023), the other coefficients demonstrated good to excellent reliability, with values over .85. The only tool for which McDonald's omega could not be calculated because of negative correlations among some items was DERS-SF (Kaufman et al., 2016). Negative correlations were found for the Awareness subscale; a similar issue has previously been reported and the exclusion of items comprising this subscale may be suggested (e.g., Fowler et al., 2014; Gouveia et al., 2022; Moreira et al., 2022). Therefore we repeated the mediation analyses excluding the items comprising the Awareness subscale. The obtained results remained unchanged –detailed results are provided in the Supplementary material. Due to an excellent alpha value and successful use of the Polish version of the tool in previous studies (e.g., Gambin et al., 2023; Pankowski et al., 2023; Woźniak‐Prus et al., 2023), we decided to use the questionnaire in its full, unchanged form for our main analysis reported in the current work.

### Statistical Analysis

In the first step, we analyzed bivariate correlations between all analyzed variables and descriptive statistics. Next, we conducted mediation analyses, in which attachment avoidance and anxiety served as simultaneous predictors, ER difficulties as a mediating variable, and CSBD or PPU symptom severity as the dependent variable. We adjusted for sociodemographic characteristics and the frequency of various sexual behaviors. We created three models depending on the measure used to assess CSBD: BPS, CSBD-19 and CSBD-DI.

For mediation models, effects were evaluated using bootstrapping with 5000 iterations. We tested the strength of the analyzed effects with 95% biased-corrected bootstrapped confidence intervals.

We used the Statistical Package for the Social Sciences (SPSS, IBM Corp., v.27, 2020) to perform correlation analyses. For mediation analyses, we used the Amos software (Arbuckle, 2011).

As we predicted that the two attachment insecurity dimensions may be associated with different kinds of sexual behaviors, we created linear regression models in which attachment avoidance and attachment anxiety served as independent variables and the frequency of pornography use, masturbation, and sexual intercourse were treated as dependent variables. We also adjusted for difficulties with ER, gender, age, sexual orientation, and relationship status. We also conducted moderation analyses in which ER difficulties served as a moderator of the relationships between attachment anxiety/avoidance and CSBD/PPU symptom severity. We reported the results of analyses conducted for the DERS-SF general score. Additionally, we also conducted the calculations for the model without the Awareness subscale, due to the reliability problems related to the scale reported by other researchers, which was discussed in the main text of the manuscript (e.g., Fowler et al., 2014; Gouveia et al., 2022; Moreira et al., 2022). However, for all conducted models, the results of the moderation did not reach statistical significance. In other words, ER difficulties did not significantly moderate the relationship between attachment dimensions and CSBD and/or PPU symptom severity. For moderation analyses, we used SPSS (SPSS, IBM Corp., v.27, 2020). Due to issues with McDonald’s omega for the DERS-SF’s (Kaufman et al., 2016) and negative correlations of the Awareness subscale with other subscales, we also conducted mediation analyses excluding this subscale, which did not result in notable changes to the obtained results. As these analyses are not the focus of the current manuscript, we included these models in the Supplementary material.

## Results

### Descriptive Statistics and Correlation Coefficients

Descriptive statistics (means, SDs, and ranges), and correlation indices (Pearson’s *r*) between the variables included in the analysis are presented in Table 1.

**Table 1:** Descriptive statistics and correlation indices (Pearson’s *r*) estimating the strengths of relationships between variables

	*M* (*SD*)	Range	1	2	3	4	5	6	7	8	9	10
1. Age (in years)	50.49 (13.32)	20.00–72.00	–									
2. Frequency of pornography use	2.88 (2.34)	.00–8.00	− .20^**^	–								
3. Frequency of masturbation	2.94 (2.42)	.00–8.00	− .25^**^	.72^**^	–							
4. Frequency of sexual intercourse	4.16 (2.43)	.00–8.00	− .18^**^	.10^**^	.05	–						
5. CSBD severity (CSBD-19 General Score)	32.86 (10.12)	19.00–76.00	− .06	.34^**^	.27^**^	.10^**^	–					
6. CSBD severity (CSBD-DI General Score)	.35 (.97)	.00–7.00	− .15^**^	.27^**^	.27^**^	.04	34^**^	–				
7. PPU severity (BPS General Score)	1.63 (2.37)	.00–10.00	− .09^**^	.51^**^	.40^**^	− .03	.52^**^	43^**^	–			
8. Attachment avoidance	2.88 (1.04)	1.00–6.63	− .09^**^	.07^*^	.08^*^	− .27^**^	.17^**^	.11^**^	.14^**^	–		
9. Attachment anxiety	3.39 (1.37)	1.00–7.00	− .15^**^	.12^**^	.15^**^	− .19^**^	.27^**^	.20^**^	.28^**^	.29^**^	–	
10. ER difficulties (DERS General Score)	2.03 (0.56)	1.00–4.33	− .22^**^	.10^**^	.11^**^	− .14^**^	.35^**^	.20^**^	.31^**^	.28^**^	.46^**^	–

^*^p < .05 ^**^p < .001

ER—emotion regulation; CSBD—compulsive sexual behavior disorder; PPU—problematic pornography use; CSBD-19—Compulsive Sexual Behavior Disorder Scale; CSBD-DI—Compulsive Sexual Behavior Disorder-Diagnostic Inventory; DERS—Difficulties in Emotion Regulation Scale

### Mediation Analyses

**Fig. 2:**
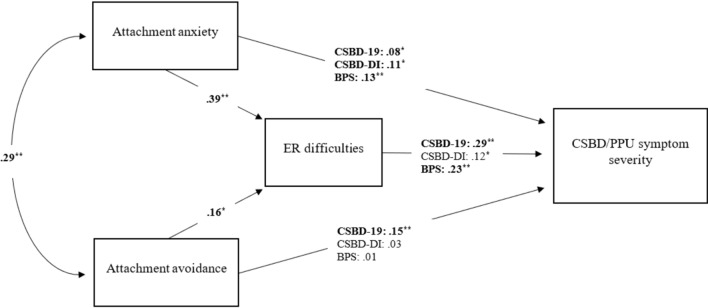
Mediation model of relationships between attachment anxiety, attachment avoidance, and compulsive sexual behavior disorder or problematic pornography use with standardized coefficients. ^*^p < .05 ^**^p < .001. Note ER—emotion regulation; CSBD—compulsive sexual behavior disorder; PPU—problematic pornography use

Next, we performed an analysis to test whether emotion regulation difficulties (ER difficulties) mediate the relationship between insecure attachment dimensions (anxiety and avoidance) and CSBD or PPU symptom severity (for a conceptual diagram, see Fig. 2). We adjusted for the effect of gender, age, sexual orientation, relationship status (we controlled for their effect on ER difficulties, and CSBD symptom severity), and the effects of frequency of sexual behavior (pornography use, masturbation, and sexual intercourse) on CSBD and PPU. To assess the effect size of the indirect effect we used Cohen’s measure (1988) as a reference standard, which may be used to assess the amount of mediation (Shrout & Bolger, 2002), however, we squared the values as an indirect effect is the product of two effects (from 0.01 to 0.09—small effect size, from 0.09 to 0.25—moderate, more than 0.25—large effect size) (Kenny, 2021).

We created two separate mediation models for CSBD severity depending on the measure used to assess the level of the dependent variable CSBD-19 (Bőthe et al., 2020a) or CSBD-DI (Grubbs et al., 2023)*.*

For all three models, attachment avoidance and attachment anxiety showed a moderate positive correlation (r = .29, *p* < .001, 95%CI_bc_[.22, .36]). Moreover, both attachment anxiety (*β* = .39, *p* < .001, 95%CI_bc_[.33, .45]) and attachment avoidance (*β* = .16, *p* < .05, 95%CI_bc_[.09, .21]) predicted greater ER difficulties.

#### Compulsive Sexual Behavior Disorder

**CSBD-19**. In the model presented in Table 2, we placed CSBD symptom severity measured by CSBD-19 (Bőthe et al., 2020a) in the role of the dependent variable. The model showed a satisfactory fit to the data (*χ*^2^(3) = 6.93, *p* = .074; RMSEA = .036; SRMR = .007; and CFI = .999).When placed in the role of simultaneous predictors both attachment anxiety (*β* = .15, *p* < .001, 95%CI_bc_[.08, .22]) and avoidance (*β* = .08, *p* < .05, 95%CI_bc_[.02, .14]) significantly predicted CSBD symptom severity. Moreover, both these effects were partially mediated by emotion regulation difficulties; the indirect effects on CSBD: (1) attachment anxiety: *β* = .12, *p* < .001, 95%CI_bc_[.09, .15], moderate partial mediation; (2) attachment avoidance *β* = .05, *p* < .001 95%CI_bc_[.03, .07], small partial mediation. Emotion regulation difficulties also had a direct positive effect on CSBD symptom severity (*β* = .29, *p* < .001, 95%CI_bc_[.23, .36]).

**Table 2:** Statistical mediation model of relationships between attachment anxiety, attachment avoidance, and compulsive sexual behavior disorder symptoms (measured by the Compulsive Sexual Behavior Disorder Scale [CSBD-19])

Independent variable	Type of effect	Effect path	*β*	95% C.I		*p*	Interpretation
				Lower	Upper		
Attachment anxiety	Indirect	Attachment anxiety → ER difficulties → CSBD	.12	.09	.15	< .001	Moderate partial mediation
	Direct	Attachment anxiety → CSBD	.15	.08	.22	< .001	
	Total	Attachment anxiety → CSBD	.27	.21	.33	< .001	
Attachment avoidance	Indirect	Attachment avoidance → ER difficulties → CSBD	.05	.03	.07	< .001	Weak partial mediation
	Direct	Attachment avoidance → CSBD	.08	.02	.14	.011	
	Total	Attachment avoidance → CSBD	.12	.06	.18	< .001	

Emotion regulation difficulties were included in the models in the mediator role

Entries are standardized coefficients. Models controlled for the effects of gender, age, relationship status, sexual orientation, and frequency of pornography watching, masturbation, and sexual intercourse

ER—emotion regulation; CSBD—compulsive sexual behavior disorder symptom severity

We also found a few significant effects of variables we adjusted for in the analysis. Men were more likely to have lower ER difficulties (*β* = −.06, *p* < .05, 95%CI_bc_[−.11, −.01], as well as higher CSBD symptom severity (*β* = .16, *p* < .05, 95%CI_bc_[.09, .22]). Moreover, a higher age was associated with lower ER difficulties (*β* = −.15, *p* < .001, 95%CI_bc_[−.21, −.09]), but with higher CSBD symptom severity (*β* = .10, *p* < .05, 95%CI_bc_[.04, .16]). Both being in a formal (*β* = .13, *p* < .05, 95%CI_bc_[.05, .21]) and informal relationship (*β* = .10, *p* < .05, 95%CI_bc_[.02, .18]) was predictive of higher CSBD symptom severity. Additionally, being sexually diverse was associated with higher CSBD symptoms (*β* = −.08, *p* < .05, 95%CI_bc_[−.14, −.02]. A higher frequency of porn watching (*β* = .17,* p* < .001, 95%CI_bc_[.09, .26]) and sexual intercourse (*β* = .12, *p* < .05, 95%CI_bc_[.05, .18]) was a risk factor for higher CSBD symptom severity.

**CSBD-DI**. In the model presented in Table 3, CSBD symptom severity measured by the CSBD-DI (Grubbs et al., 2023) was the dependent variable. The model showed a satisfactory fit to the data (*χ*^2^(3) = 6.93, *p* = .074; RMSEA = .036; SRMR = .007; and CFI = .999). When placed in the role of simultaneous predictors, attachment anxiety (*β* = .11, *p* < .05, 95%CI_bc_[.03, .18]), but not avoidance (*β* = .03, *p* = .427, 95%CI_bc_[−.05, .14]) significantly predicted CSBD symptom severity. Moreover, the effect of attachment anxiety on CSBD symptom severity was partially mediated by emotion regulation difficulties (*β* = .05, *p* < .05, 95%CI_bc_[.02, .08], small partial mediation). The effect of attachment avoidance on CSBD symptom severity was fully mediated by ER difficulties (indirect effect: *β* = .02, *p* < .05, 95%CI_bc_[.01, .04], small effect size). Difficulties with ER had direct positive effect on CSBD symptoms (*β* = .12, *p* < .05, 95%CI_bc_[.04, .19]).

**Table 3:** Statistical mediation model of relationships between attachment anxiety, attachment avoidance, and compulsive sexual behavior disorder symptoms (measured by the Compulsive Sexual Behavior Disorder-Diagnostic Inventory [CSBD-DI])

Independent variable	Type of effect	Effect path	*β*	95% C.I		*p*	Interpretation
				Lower	Upper		
Attachment anxiety	Indirect	Attachment anxiety → ER difficulties → CSBD	.05	.02	.08	.002	Weak partial mediation
	Direct	Attachment anxiety → CSBD	.11	.03	.18	.004	
	Total	Attachment anxiety → CSBD	.15	.07	.22	.001	
Attachment avoidance	Indirect	Attachment avoidance → ER difficulties → CSBD	.02	.01	.04	.001	Weak full mediation
	Direct	Attachment avoidance → CSBD	.03	− .05	.14	.427	
	Total	Attachment avoidance → CSBD	.05	− .03	.15	.224	

Emotion regulation difficulties were included in the models in the mediator role

Entries are standardized coefficients. Models controlled for the effects of gender, age, relationship status, sexual orientation, and frequency of pornography watching, masturbation, and sexual intercourse

ER—emotion regulation; CSBD—compulsive sexual behavior disorder symptom severity

We also found a few significant effects of variables we adjusted for in the analysis. Male gender was predictive of lower ER difficulties (*β* = −.06, *p* < .05, 95%CI_bc_[−.11, −.01]. Moreover, higher age was associated with lower ER difficulties (*β* = −.15, *p* < .001, 95%CI_bc_[−.21, −.09]). A higher frequency of porn watching (*β* = .12, *p* < .05, 95%CI_bc_[.03, .22]) and masturbation (*β* = .13, *p* < .05, 95%CI_bc_[.04, .20]) was a risk factor for higher CSBD symptom severity.

#### Problematic Pornography Use

We created a model with the same predictors, but with PPU symptom severity (measured by the BPS; Kraus et al., 2020) serving as the dependent variable (Table 4). The model showed a satisfactory fit to the data (*χ*^2^(3) = 6.93, *p* = .074; RMSEA = .036; SRMR = .007; and CFI = .999). When placed in the role of simultaneous predictors, only attachment anxiety (*β* = .13, *p* < .001, 95%CI_bc_[.08, .19]), but not avoidance (*β* = .01, *p* = .805, 95%CI_bc_[−.04, 0.06]) significantly predicted PPU symptom severity. Moreover, the effect of attachment anxiety was partially mediated by emotion regulation difficulties (the indirect effect on PPU for attachment anxiety: *β* = .09, *p* < .001, 95%CI_bc_[.06, .12], moderate effect size). The influence of attachment avoidance on PPU symptom severity was fully mediated by ER difficulties *β* = .04, *p* < .001, 95%CI_bc_[.02, .06], with a small effect size. Emotion regulation difficulties showed a direct positive effect on PPU symptom severity (*β* = .23, *p* < .001, 95%CI_bc_[.17, .29]).

**Table 4:** Statistical mediation model of relationships between attachment anxiety, attachment avoidance, and problematic pornography use symptoms

Independent variable	Type of effect	Effect path	*β*	95% C.I		*p*	Interpretation
				Lower	Upper		
Attachment anxiety	Indirect	Attachment anxiety → ER difficulties → PPU	.09	.06	.12	< .001	Moderate partial mediation
	Direct	Attachment anxiety → PPU	.13	.08	.19	< .001	
	Total	Attachment anxiety → PPU	.22	.17	.28	< .001	
Attachment avoidance	Indirect	Attachment avoidance → ER difficulties → PPU	.04	.02	.06	< .001	Weak full mediation
	Direct	Attachment avoidance → PPU	.01	− .04	.06	.805	
	Total	Attachment avoidance → PPU	.04	− .01	.09	.118	

Emotion regulation difficulties were included in the models in the mediator role. Entries are standardized coefficients

Entries are standardized coefficients. Models controlled for the effects of gender, age, relationship status, sexual orientation, and frequency of pornography watching, masturbation, and sexual intercourse

ER—emotion regulation; PPU—problematic pornography use symptom severity

Moreover, male gender was predictive of milder ER difficulties (*β* = −.06, *p* < .05, 95%CI_bc_[−11, −.01]) and higher PPU symptom severity (*β* = .11, *p* < .05, 95%CI_bc_[.05, .17]). Additionally, higher age was associated with fewer ER difficulties (*β* = −.15, *p* < .001, 95%CI_bc_[−.21, −.09]). Regarding sexual activity, only the frequency of pornography use predicted more severe PPU symptoms (*β* = .40, *p* < .001, 95%CI_bc_[.32, .49]).

## Discussion

To the best of our knowledge, our study was the first to investigate the associations between attachment insecurity dimensions and CSBD and PPU, employing difficulties with ER as the explanatory mechanism. Our results are in line with previous studies showing the importance of attachment insecurity (see Efrati et al., 2022) and ER difficulties in CSBD (e.g., Engel et al., 2019; Hashemi et al., 2018; Pachankis et al., 2014). Moreover, they corroborate the theoretical claim that ER difficulties may be a useful framework for explaining the impact of attachment insecurity on CSBD (see Lew-Starowicz et al., 2020).

### Attachment Insecurity and Compulsive Sexual Behavior Disorder

The results regarding the direct effects of attachment insecurity dimensions on CSBs are mixed (H1 and H3 were supported, H2 was partially corroborated, H4 was not supported). However, the indirect effect (mediated by ER difficulties) of attachment insecurity dimensions on CSBs was significant for all tested paths. It means that the effect of attachment avoidance on PPU was fully encapsulated by the mediator variable—difficulties with ER.

Our results clearly show the associations between attachment insecurity and CSBs, in line with previous research (e.g., Ciocca et al., 2021; Gilliland et al., 2015), presumably showing the greater importance of attachment anxiety in explaining CSBD and PPU symptom severity. Prior research regarding the role of specific attachment insecurity dimensions in CSBD has shown inconsistent results. Some studies show that the differences in CSBD symptom severity are better explained by attachment anxiety (Beutel et al., 2017; Giordano et al., 2017; Kircaburun et al., 2023). However, other studies point to attachment avoidance being a stronger predictor of CSBD than attachment anxiety (Crocker, 2015; Varfi et al., 2019).

The inconsistency regarding the relative importance of specific attachment insecurity dimensions across different research may partly stem from a lack of controlling for the specific types of assessed CSBD. Different attachment insecurity dimensions are reflected in specific difficulties in bonding with others that could presumably be associated with, for example, engagement in different kinds of online and offline sexual activities (Varfi et al., 2019). For instance, adolescents who engaged only in online sexual behavior displayed greater attachment avoidance than those who engaged in both online and offline sexual activities (Efrati & Amichai-Hamburger, 2021). Presumably, individuals characterized by high attachment avoidance may at least in some circumstances prefer activities that do not require direct interactions with others (to prevent bonding with others) and would more often get involved in solitary sexual activities in a virtual space. On the other hand, high attachment anxiety could be associated with more frequent dyadic activities requiring direct contact with sexual partners. This hypothesis, however, does not seem to be fully supported by the additional analyses that we conducted (see Table S1 in Supplementary material). Similarly, attachment avoidance and anxiety predicted less frequent dyadic sexual activity which may be explained by different motifs; attachment avoidance was associated with undertaking dyadic sexual activity in order to fulfill sexual needs, while attachment anxiety was related to striving for the fulfilment of emotional needs (Saint‐Eloi Cadely et al., 2020).

### Emotion Regulation and Compulsive Sexual Behavior Disorder

Our results show the direct positive effect of ER difficulties on CSBD and PPU symptom severity (corroborating hypotheses H5 and H6), which is in line with previous empirical studies (Hashemi et al., 2018; Pachankis et al., 2014). It is important to highlight, however, that the above-cited, as well as many other previous studies (e.g., Engel et al., 2019; Reid et al., 2008, 2011), conceptualized CSBD as a hypersexual disorder [HD; proposed for but ultimately not included in the DSM-5 (Kafka, 2010)]. One of the diagnostic criteria for HD was engagement in sexual behaviors in order to cope with dysphoric mood states. The current conceptualization of CSBD, as well as the instruments we used for its assessment, are based on the ICD-11 criteria (WHO, 2022a) that do not include this criterion. Such a difference in conceptualizations may affect the strength of the relationship between ER difficulties and symptom severities. Nonetheless, to the best of our knowledge, our study is the first to use this conceptualization to show the associations between CSBD and ER difficulties. Assessment of PPU differs in this regard as well, for example, the BPS (Kraus et al., 2020) (that we used to assess PPU) has an item that reflects pornography use as a maladaptive coping strategy. This may result in stronger associations between PPU symptom severity and ER difficulties. The relationship between PPU symptom severity and ER difficulties is, however, also supported by studies that use measures that do not reflect this aspect of PPU (Baranowski et al., 2019; Laier & Brand, 2017).

### Emotion Regulation as a Mechanism Explaining the Relationship Between Attachment Insecurity and Compulsive Sexual Behavior Disorder

Our results show that ER difficulties may act as a mechanism explaining the relationship between attachment insecurity and CSBD and PPU. In our study, the relationships between both attachment anxiety and avoidance on one side, and CSBD or PPU on the other, were all mediated by difficulties with ER (these results lend support to H7-H10). Moreover, in the case of the associations between attachment avoidance and PPU symptom severity, and between attachment avoidance and CSBD (as measured by CSBD-DI, but not CSBD-19), they were completely mediated by ER. This means that the effect of attachment avoidance on PPU and CSBD severity (when assessed by CSBD-DI) is fully encapsulated by difficulties with ER. The difference in those results may stem from the different construction of the two measures; we elaborate on this topic in the next section (Assessment of CSBD/PPU symptom severity with CSBD-19 vs. CSBD-DI).

One of the possible interpretative routes for the results of our mediation analyses underlines that attachment avoidance is associated with deactivating ER strategies. Attachment avoidance is characterized by fear of intimacy, interdependence, and emotional openness (Crowell et al., 2008). In order to avoid those aspects of bonding to others, the individual undertakes efforts to inhibit their emotions so their attachment system is deactivated. In that way, compulsive sexual behaviors can potentially act as a substitute for intimate relationships and lack of intimacy (Leedes, 1999a, 2001).

Anxiously attached individuals, however, have been taught to intensify their negative emotions to attract the attention of attachment figures. They are characterized by fear of insufficient love or abandonment and may engage in sexual activity to alleviate distress resulting from those beliefs (Crowell et al., 2008). Engagement in compulsive sexual behaviors, however, may further intensify those feelings, for example, lack of emotional engagement of a casual sexual partner may be interpreted by a highly anxious individual as abandonment and this may further intensify the fear of abandonment in future interactions.

These mechanisms may probably determine engagement in different types of sexual behaviors depending on which insecure attachment dimension is more prominent for an individual. The models in which we checked how the specific attachment insecurity dimensions predict the frequency of different sexual behaviors provide some explanation for those results (see Supplementary Material). Attachment avoidance, as well as attachment anxiety, predicted lower sexual intercourse frequency. On the other hand, only attachment anxiety predicted a higher frequency of solitary sexual behaviors, while attachment avoidance showed no association with them. The correlation indices for the relationships with the frequency of pornography use and masturbation for both dimensions are, however, all negative and significant. Attachment avoidance, compared to attachment anxiety, may therefore indicate generally lower engagement in sexual behaviors. These results are, however, preliminary and limited, thus this hypothesis should be disentangled in future studies.

Detailed assessment of specific sexual activities and motives for their undertaking, as well different aspects of insecure attachment, is needed in future studies to disentangle these complex associations. For example, sexual activity may be seen as a means of fulfilling sexual needs and may be associated with pleasure maximization, as well as having a higher number of sexual partners for highly avoidant individuals (Szielasko et al., 2013). On the other hand, for individuals high in attachment anxiety engagement in sexual activities in order to satisfy their emotional needs may be more prominent (Saint‐Eloi Cadely et al., 2020). This claim seems consistent with consideration of CSBD in terms of an attachment disorder (Flores, 2004; Gilliland et al., 2015; Riemersma & Sytsma, 2013; Zapf et al., 2008).

To sum up, to the best of our knowledge, our study is the first to show that ER difficulties may mediate the relationship between attachment insecurity and, CSBD and PPU.

### Assessment of Compulsive Sexual Behavior Disorder and Problematic Pornography Use Symptom Severity with Compulsive Sexual Behavior Disorder-19 vs. Compulsive Sexual Behavior Disorder-Diagnostic Inventory

It is important to note the possible explanation for the difference between the results of mediation analyses regarding CSBD depends on the measures used. Both measures of CSBD that we used are based on the current CSBD’s ICD-11 criteria. However, the design of both measures is significantly different, with CSBD-DI using statements with a higher degree of complexity and different approach to the response scale and response grading. A descriptive analysis (Table 1) shows that, perhaps due to this complexity, research participants seemed to be more conservative in assessing their CSBD symptoms using CSBD-DI than CSBD-19 (mean response for CSBD-DI: *M* = .35, *SD* = .97, possible range between 0 and 7 points; mean response for CSBD-19: *M* = 32.86, *SD* = 10.12, possible range between 19 and 76 points). Associations between CSBD-DI on one side and attachment and emotion regulation variables on the other side may have been slightly diminished due to floor effects for CSBD-DI, which were not present for CSBD-19. However, the obtained inconsistency needs to be further explored in future studies to confirm whether the measures can be used interchangeably in general population research and what the possible differences between them are. Importantly, our study did not involve a clinical sample and our conclusions do not extend to CSBD-DI and CSBD-19 performance in clinical research, which should also be addressed in future studies. Due to concerns about overestimation of CSBD/PPU occurrence (Lewczuk et al., 2023b; Lew-Starowicz & Coleman, 2022), a conservative approach may even be preferable in aiding CSBD diagnosis, however, future research including clinical samples should further investigate this subject.

### Additional Variables

In our study, the male gender was associated with fewer ER difficulties, however, the effect size of the relationship was small. This result is inconsistent with previous research showing no gender differences (Giromini et al., 2017; Sörman et al., 2022; Westerlund & Santtila, 2018) in difficulties with ER. Further, higher age was associated with fewer ER difficulties in line with previous studies (Giromini et al., 2017; Westerlund & Santtila, 2018).

Contrary to the existing evidence (e.g., Grubbs et al., 2019; Lewczuk et al., 2023b, 2023c) age showed no significant relationship to PPU or CSBD (as measured by the CSBD-DI, and a negative relationship when assessed with the CSBD-19). This can be explained by the unrepresentativeness of the current sample in terms of sociodemographic characteristics and participants’ lower mean age compared to the previously cited studies. Male gender, similar to the results of previous studies (e.g., Bőthe et al., 2020a, 2020b; Kraus et al., 2020), was linked to higher severity of PPU and CSBD, however, the underrepresentation of female samples in research should be noted (see e.g., Kowalewska et al., 2020, 2022). Being in a romantic relationship was associated with higher CSBD symptoms severity (but only when measured with CSBD-19). The results regarding the impact of relationship status on CSBD and PPU are inconsistent (no significant relationship, e.g., Lewczuk et al., 2023c; positive predictor, e.g., Wizła et al., 2022). The pattern of bonding to others that the attachment insecurity describes may play a more prominent role in CSBD and PPU symptom severity rather than the relationship status itself. Thus, the effect of attachment insecurity may have encapsulated the relationship status’ effect on CSBD and PPU. Non-heterosexual orientation was associated with higher CSBD symptom severity, which is consistent with previous research (Dickenson et al., 2018; see also Black, 2000; Bőthe et al., 2018; Kelly et al., 2009; Parsons et al., 2013, 2015). This result can be linked to the minority stress experienced by sexually diverse individuals that is associated with CSBD and PPU symptoms (Lewczuk et al., 2023a). The results of our study regarding the relationships between the frequency of specific sexual activities and CSBD/PPU symptoms are inconsistent, lending support to the claim that a high frequency of sexual behaviors cannot be unequivocally associated with CSBD (e.g., Bőthe et al., 2020a, 2020b; Carvalho et al., 2015).

### Limitations and Future Directions

A few limitations to our study should be noted. The study’s cross-sectional design does not allow for causal inference; hence the conclusions should be treated with caution. Future longitudinal studies should help disentangle and confirm the causal character of the relationships obtained in our study. Research would also benefit from an experimental design that would encompass psychotherapeutic interventions aimed at modifying ER strategies or attachment styles and how changing those aspects may affect CSBD and PPU symptom severity. Future studies could also concentrate on specific maladaptive ER strategies, and not general ER difficulties. Our conclusions are limited to a convenience sample from the Polish adult population. Future studies should check if the results replicate in representative samples, different cultural contexts, and more vulnerable groups such as sexual minorities and adolescents. Research regarding attachment insecurity’s role in CSBD would also benefit from distinguishing between different kinds of online and offline sexual activities as they may be driven by specific mechanisms (Efrati & Amichai-Hamburger, 2021). Another issue to consider is the context of the Covid-19 pandemic that might have affected the results of the present study, as the impact of this context on CSBD/PPU symptoms is inconsistent (Bőthe et al., 2022; Grubbs et al., 2022; Koós et al., 2022; Xiong et al., 2020; Zattoni et al., 2020). Therefore, future studies should aim to replicate our results when the epidemic situation is different.

### Implications

Our study has both theoretical and practical implications. The importance of ER difficulties in CSBD/PPU development and maintenance should be confirmed in future longitudinal studies; such evidence would imply that effective ER should be targeted in therapeutic interventions for CSBD (Gratz et al., 2015) and that individuals experiencing CSBD may benefit from therapeutic approaches focused on ER regulation, for example, from dialectical behavior therapy (see Harvey et al., 2019). Another, “third wave” cognitive behavioral therapy approach that may be beneficial for targeting emotion dysregulation in CSBD/PPU is Acceptance Commitment Therapy which emphasizes the role of experiential avoidance [attempts to avoid and escape one's internal experiences, among others, emotions (Hayes et al., 1996)] in the development and maintenance of psychiatric problems [among them CSBD (Borgogna & McDermott, 2018; Brem et al., 2017; Levin et al., 2019; Wetterneck et al., 2012)]. Generally, mindfulness-based approaches are associated with strategies aimed at handling emotions and those treatments have been found to be effective in decreasing ER difficulties (see Roemer et al., 2015). Another issue to highlight is the importance of therapeutic alliance and the possibility of modifying attachment styles in psychotherapy (e.g., Gidhagen et al., 2018; Kinley & Reyno, 2013; Spence et al., 2016; Travis et al., 2001; see Levy et al., 2018; Slade & Holmes, 2019).

### Conclusions

Our study is the first to show the mediating role of ER difficulties in the relationship between attachment insecurity and CSBD as well as PPU symptom severity. Overall, difficulties with ER partially mediated the effect of attachment anxiety and fully mediated the effect of attachment avoidance on CSBD and PPU. Emotion regulation difficulties can be employed as an explanatory mechanism for the associations between attachment insecurity and CSBD and PPU. Insecurely attached individuals develop maladaptive ER strategies in interactions with primary caregivers, which in turn can contribute to developing CSBD and PPU symptoms in the future. Future studies should expand on our results by investigating attachment, ER and CSBD/PPU relationships in different cultural contexts and using different methodological approaches. Moreover, therapeutic interventions targeting ER difficulties and attachment insecurity should be investigated as a promising aid for CSBD and PPU.

## Supplementary Information

Below is the link to the electronic supplementary material.

Supplementary file1 (DOC 321 KB)

## Data Availability

Not applicable.

## Footnotes

1 The provided McDonald’s omega coefficient was calculated after items comprising the Awareness subscale were excluded due to negative correlations with other items of the measure. The issue is further elaborated on at the end of the Measures section.
