# Supplementary material for: The Associations Between Attachment Insecurity and Compulsive Sexual Behavior Disorder or Problematic Pornography Use: The Mediating Role of Emotion Regulation Difficulties
Source: Arch Sex Behav. 2024 Jun 19;53(9):3419–36. doi: 10.1007/s10508-024-02904-7 (PMC11390895; doi:10.1007/s10508-024-02904-7)
Supplement: Supplementary file 1 — Supplementary file1 (DOC 321 KB) [file 10508_2024_2904_MOESM1_ESM.doc]

**Supplementary material**

**Moderation analyses**

**Figure S1**

*Conceptual diagram: moderation model of relationships between attachment anxiety, attachment avoidance, and compulsive sexual behavior disorder or problematic pornography use.*

*
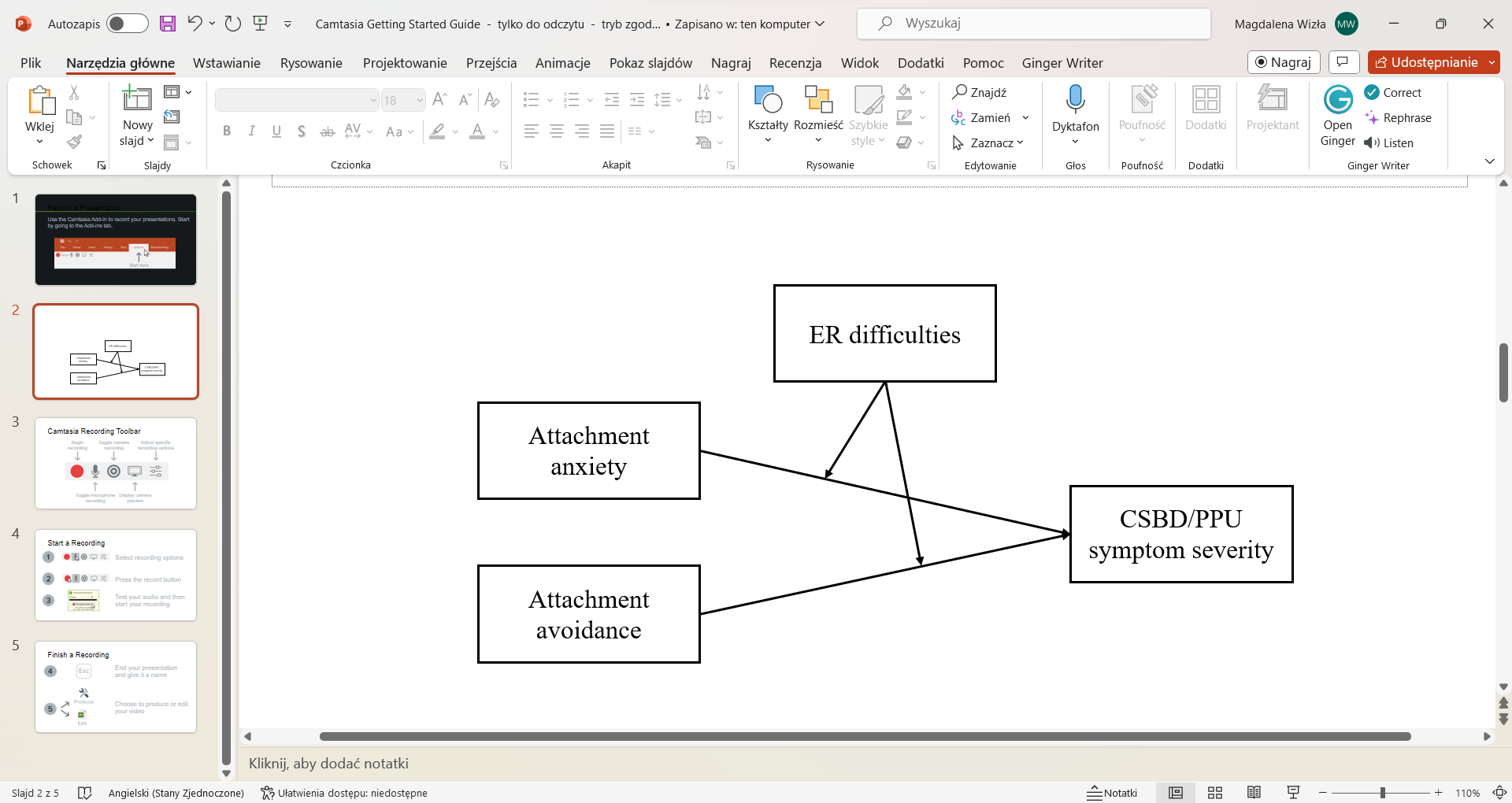
*

*Note. ER* – *emotion regulation; CSBD* – *compulsive sexual behavior disorder; PPU* – *problematic pornography use*

**Table S1**

*Emotion regulation difficulties as a moderator of the relationship between attachment anxiety or avoidance and compulsive sexual behavior disorder or problematic pornography use.*

|  | CSBD-19 general score | | | | CSBD-DI general score | | | | BPS general score | | | |
| --- | --- | --- | --- | --- | --- | --- | --- | --- | --- | --- | --- | --- |
| Predictor | *B* [CI 95%] | *SE* | *p* | *∆R* | *B* [CI 95%] | *SE* | *p* | *∆R* | *B* [CI 95%] | *SE* | *p* | *∆R* |
| Attachment anxiety | 1.10 [.65, 1.56] | .23 | <.000 |  | .07 [.03, .12] | .02 | .003 |  | .24 [.14, .34] | .05 | <.001 |  |
| Attachment avoidance | .75 [.19 1.32] | .29 | .009 |  | .03 [-.03, .09] | .03 | .329 |  | .03 [-.10, .15] | .06 | .680 |  |
| ER difficulties | 5.25 [4.08, 6.42] | .60 | <.001 |  | .21 [.09, .34] | .06 | .001 |  | .89 [.63, 1.15] | .13 | <.001 |  |
| Attachment anxiety x ER difficulties | .18 [.48, .84] | .34 | .589 | .00 | -.02 [-.10, .05] | .04 | .490 | .00 | .13 [-.02, .27] | .07 | .092 | .00 |
| Attachment avoidance x ER difficulties | -0,27 [-.1.19, .65] | .47 | .567 | .00 | .01 [-.09, .11] | .05 | .824 | .00 | .01 [-.19, .22] | .10 | .913 | .00 |
|  | *F* = 31.60 (<.001); *R^2^_adj_*= .284 | | | | *F* = 11.74 (<.001); *R^2^_adj_*= . 122 | | | | *F* = 43.98 (<.001); *R^2^_adj_*= .358 | | | |

*Note. The variables were centered prior to analysis. Models were controlled for the effects of gender, age, relationship status, sexual orientation, and frequency of pornography watching, masturbation, and sexual intercourse*

*Note 2. CSBD-19* – *Compulsive Sexual Behavior Disorder Scale; CSBD-DI* – *Compulsive Sexual Behavior Disorder – Diagnostic Inventory; BPS* – *Brief Pornography Screen; ER* – *emotion regulation*

**Mediation Analyses**

In Tables S2-S4 we present the results of mediation analyses in which we excluded the ***Awareness*** subscale from the general score of the Difficulties in Emotion Regulation Scale to reflect ER difficulties.

For all three models, attachment avoidance and attachment anxiety showed a moderate positive correlation (r=.29, *p*<.001, 95%CI_bc_[.22,.36]). Moreover, both attachment anxiety (*β*=.40, *p*<.001, 95%CI_bc_[.34,.46]) and attachment avoidance (*β* =.10, *p*<.05, 95%CI_bc_[.04,.17]) predicted greater ER difficulties.

***Compulsive sexual behavior disorder***

**CSBD-19.** In the model presented in Table S2, we placed CSBD symptom severity measured by CSBD-19 (Bőthe, Potenza, et al., 2020) in the role of the dependent variable. The model showed a satisfactory fit to the data (*χ^2^*(3)=7.25, *p*=.064; RMSEA = .038; SRMR = .007; and CFI = .999). When placed in the role of simultaneous predictors both attachment anxiety (*β*=.15, *p*<.001, 95%CI_bc_[.08,.22]) and avoidance (*β*=.10, *p*<.05, 95%CI_bc_[.10,.19]) significantly predicted CSBD symptom severity. Moreover, both these effects were partially mediated by emotion regulation difficulties; the indirect effects on CSBD: (1) attachment anxiety: *β*=.11, *p*<.001, 95%CI_bc_[.08,.15], moderate partial mediation; (2) attachment avoidance *β*=.03, *p*<.05 95%CI_bc_[.01,.05], small partial mediation. Emotion regulation difficulties also had a direct positive effect on CSBD symptom severity (*β*=.28, *p*<.001, 95%CI_bc_[.21,.34]).

We also found a few significant effects of variables we adjusted for in the analysis. Men were more likely to have lower ER difficulties (*β*=-.08, *p*<.05, 95%CI_bc_[-.13,-.03], as well as higher CSBD symptom severity (*β*=.16, *p*<.001, 95%CI_bc_[.10,.22]). Moreover, higher age was associated with lower ER difficulties (*β*=-.16, *p*<.001, 95%CI_bc_[-.22,-.10]), but with higher CSBD symptom severity (*β*=.10, *p*<.05, 95%CI_bc_[.04,.16]). Both being in a formal (*β*=.14, *p*<.001, 95%CI_bc_[.06,.21]) and informal relationship (*β*=.10, *p*<.05, 95%CI_bc_[.02,.18]) was predictive of higher CSBD symptom severity. Additionally, being sexually diverse was associated with higher CSBD symptoms (*β*=-.08, *p*<.05, 95%CI_bc_[-.14,-.01]. A higher frequency of porn watching (*β*=.17, *p*<.001, 95%CI_bc_[.09,.26]) and sexual intercourse (*β*=.12, *p*<.05, 95%CI_bc_[.05,.19]) was a risk factor for higher CSBD symptom severity.

**CSBD-DI.** In the model presented in Table S3, CSBD symptom severity measured by the CSBD-DI (Grubbs et al., 2023) was the dependent variable. The model showed a satisfactory fit to the data (*χ^2^*(3)=7.25, *p*=.064; RMSEA = .038; SRMR = .007; and CFI = .998). When placed in the role of simultaneous predictors, attachment anxiety (*β*=.11, *p*<.05, 95%CI_bc_[.03,.18]), but not avoidance (*β*=.04, *p*=.339, 95%CI_bc_[-.05,.14]) significantly predicted CSBD symptom severity. Moreover, the effect of attachment anxiety on CSBD symptom severity was partially mediated by emotion regulation difficulties (*β*=.04, *p*<.05, 95%CI_bc_[.01,.08], small partial mediation). The effect of attachment avoidance on CSBD symptom severity was fully mediated by ER difficulties (indirect effect: *β*=.02, *p*<.05, 95%CI_bc_[.00,.03], small effect size). Difficulties with ER had a direct positive effect on CSBD symptoms (*β*=.12, *p*<.05, 95%CI_bc_[.04,.19]).

We also found a few significant effects of variables we adjusted for in the analysis. Male gender was predictive of lower ER difficulties (*β*=-.08, *p*<.05, 95%CI_bc_[-.13,-.03]. Moreover, higher age was associated with lower ER difficulties (*β*=-.16, *p*<.001, 95%CI_bc_[-.22,-.10]). A higher frequency of porn watching (*β*=.12, *p*<.05, 95%CI_bc_[.03,.22]) and masturbation (*β*=.13, *p*<.05, 95%CI_bc_[.05,.21]) was a risk factor for higher CSBD symptom severity.

***PPU***

We created a model with the same predictors, but with PPU symptom severity (measured by the BPS; Kraus et al., 2020) serving as the dependent variable (Table S4). The model showed a satisfactory fit to the data (*χ^2^*(3)=7.25, *p*=.064; RMSEA = .038; SRMR = .007; and CFI = .999). When placed in the role of simultaneous predictors, only attachment anxiety (*β*= .13, *p*<.001, 95%CI_bc_[.07,.19]), but not avoidance (*β*=.02, *p*=.498, 95%CI_bc_[-.04,.07]) significantly predicted PPU symptom severity. Moreover, the effect of attachment anxiety was partially mediated by emotion regulation difficulties (the indirect effect on PPU for attachment anxiety: *β*=.09, *p*<.001, 95%CI_bc_[.06,.12], moderate effect size). The influence of attachment avoidance on PPU symptom severity was fully mediated by ER difficulties *β*=.02, *p*<.05, 95%CI_bc_[.01,.04], with a small effect size. Emotion regulation difficulties showed a direct positive effect on PPU symptom severity (*β*=.23, *p*<.001, 95%CI_bc_[.16,.29]).

Moreover, male gender was predictive of milder ER difficulties (*β*=-.08, *p*<.05, 95%CI_bc_[-13,-.03]) and higher PPU symptom severity (*β*=.12, *p*<.001, 95%CI_bc_[.06,.17]). Additionally, higher age was associated with fewer ER difficulties (*β*=-.16, *p*<.001, 95%CI_bc_[-.22,-.10]) and higher severity of PPU symptoms (*β*=.06, *p*<.05, 95%CI_bc_[.11,.04]). The latter of the results was the only one that differed in the current analysis when compared to the analysis including the whole DERS scale. Regarding sexual activity, only the frequency of pornography use predicted more severe PPU symptoms (*β*= .40, *p*<.001, 95%CI_bc_[.32,.48]).

**Table S2**

*Statistical mediation model of re*lationships between attachment anxiety, attachment avoidance, and compulsive sexual behavior disorder symptoms (measured by the Compulsive Sexual Behavior Disorder Scale [CSBD-19]). Emotion regulation difficulties were included in the models in the mediator role.

|  |  |  |  | | 95% C.I. | |  | |  |
| --- | --- | --- | --- | --- | --- | --- | --- | --- | --- |
| Independent variable | Type of effect | Effect path | *β* | Lower | | Upper | | *p* | Interpretation |
| Attachment anxiety | indirect | Attachment anxiety 🡪 ER difficulties 🡪 CSBD | .11 | .08 | | .15 | | <.001 | Moderate partial mediation |
|  | direct | Attachment anxiety 🡪 CSBD | .15 | .08 | | .22 | | <.001 |  |
|  | total | Attachment anxiety 🡪 CSBD | .26 | .20 | | .32 | | <.001 |  |
| Attachment avoidance | indirect | Attachment avoidance 🡪 ER difficulties 🡪 CSBD | .03 | .01 | | .05 | | .002 | Weak partial mediation |
|  | direct | Attachment avoidance 🡪 CSBD | .10 | .04 | | .15 | | .001 |  |
|  | total | Attachment avoidance 🡪 CSBD | .12 | .06 | | .18 | | .001 |  |

*Note 1 Entries are standardized coefficients. Models controlled for the effects of gender, age, relationship status, sexual orientation, and frequency of pornography watching, masturbation, and sexual intercourse.*

*Note 2 ER – emotion regulation; CSBD – compulsive sexual behavior disorder symptom severity*

**Table S3**

*Statistical mediation model of relationships between attachment anxiety, attachment avoidance, and compulsive sexual behavior disorder symptoms (measured by the Compulsive Sexual Behavior Disorde*r-Diagnostic Inventory [CSBD-DI]). Emotion regulation difficulties were included in the models in the mediator role.

|  |  |  |  | 95% C.I. | |  |  |
| --- | --- | --- | --- | --- | --- | --- | --- |
| Independent variable | Type of effect | Effect path | *β* | Lower | Upper | *p* | Interpretation |
| Attachment anxiety | indirect | Attachment anxiety 🡪 ER difficulties 🡪 CSBD | .04 | .01 | 08 | .005 | Weak partial mediation |
|  | direct | Attachment anxiety 🡪 CSBD | .11 | .03 | .18 | .004 |  |
|  | total | Attachment anxiety 🡪 CSBD | .15 | .07 | .22 | <.001 |  |
| Attachment avoidance | indirect | Attachment avoidance 🡪 ER difficulties 🡪 CSBD | .01 | .00 | .03 | .005 | Weak full mediation |
|  | direct | Attachment avoidance 🡪 CSBD | .04 | -.05 | .14 | .427 |  |
|  | total | Attachment avoidance 🡪 CSBD | .05 | -.03 | .14 | .225 |  |

*Note 1 Entries are standardized coefficients. Models controlled for the effects of gender, age, relationship status, sexual orientation, and frequency of pornography watching, masturbation, and sexual intercourse.*

*Note 2 ER – emotion regulation; CSBD – compulsive sexual behavior disorder symptom severity*

**Table S4**

*Statistical mediation model of relationships between attachment anxiety, attachment avoidance, and problematic pornography use symptoms. Emotion regulation difficulties were included in the models in the mediator role. Entries are standardized coefficients.*

|  |  |  |  | 95% C.I. | |  |  |
| --- | --- | --- | --- | --- | --- | --- | --- |
| Independent variable | Type of effect | Effect path | *β* | Lower | Upper | *p* | Interpretation |
| Attachment anxiety | indirect | Attachment anxiety 🡪 ER difficulties 🡪 PPU | .09 | .06 | .12 | <.001 | Moderate partial mediation |
|  | direct | Attachment anxiety 🡪 PPU | .23 | .13 | .33 | <.001 |  |
|  | total | Attachment anxiety 🡪 PPU | .22 | .17 | .28 | <.001 |  |
| Attachment avoidance | indirect | Attachment avoidance 🡪 ER difficulties 🡪 PPU | .02 | .01 | .04 | <.05 | Weak full mediation |
|  | direct | Attachment avoidance 🡪 PPU | .04 | -.08 | .16 | .495 |  |
|  | total | Attachment avoidance 🡪 PPU | .04 | -.01 | .09 | .135 |  |

*Note 1 Entries are standardized coefficients. Models controlled for the effects of gender, age, relationship status, sexual orientation, and frequency of pornography watching, masturbation, and sexual intercourse.*

*Note 2 ER – emotion regulation; PPU – problematic pornography use symptom severity*

**Table S5**

*Results of multivariable linear regression: attachment anxiety, attachment avoidance, and emotion regulation difficulties predicting the frequency of sexual behaviors (pornography use, masturbation, sexual intercourse)*

|  | Frequency of sexual behaviors | | | | | |
| --- | --- | --- | --- | --- | --- | --- |
|  | Pornography use | | Masturbation | | Sexual intercourse | |
|  | *β* [95% CI] | *p* | *β* [95% CI] | *p* | *β* [95% CI] | *p* |
| Attachment anxiety | **.09 [.03, .15]** | **.002** | **.10 [.04, .16]** | **.002** | -.06 [-.11, .00] | .052 |
| Attachment avoidance | -.02 [-.08, .03] | .375 | -.02 [-.08, .04] | .433 | **-.16 [-.21, -.11]** | **<.001** |
| ER difficulties | .06 [.00, .12] | .065 | .04 [-.03, .10] | .260 | -.05 [-.11, .01] | .086 |
| Gender | **.53 [.48, .58]** | **<.001** | **.37 [.31, .42]** | **<.001** | **.06[.02, .11]** | **.010** |
| Age | **-.20 [-.25, -.14]** | **<.001** | **-.23 [-.29, -.17]** | **<.001** | **-.21 [-.27, -.16]** | **<.001** |
| Sexual orientation | **-.06 [-.12, -.01]** | **.016** | **-.08 [-.13, -.02]** | **.008** | .01 [-.04, .06] | .795 |
| Formal relationship | -.02 [-.08, .04] | .555 | **-.11 [-.17, -.04]** | **.003** | **.57 [.51, .63]** | **<.001** |
| Informal relationship | **.07 [.01, .14]** | **.020** | .02 [-.05, .09] | .518 | **.53 [.47, .59]** | **<.001** |
| *F* | **62.56 (<.001)** | | **36.63 (<.001)** | | **80.29 (<.001)** | |
| *R^2^_adj_* | **.330** | | **.222** | | **.388** | |

*Note. ER – emotion regulation; Sexual orientation (0 – sexually diverse, 1 – heterosexual); Gender (0 – woman, 2 – man), Relationship status – dummy coded (reference group – single)*
